# Characterization of human FCRL4-positive B cells

**DOI:** 10.1371/journal.pone.0179793

**Published:** 2017-06-21

**Authors:** Michel Jourdan, Nicolas Robert, Maïlys Cren, Coraline Thibaut, Christophe Duperray, Alboukadel Kassambara, Michel Cogné, Karin Tarte, Bernard Klein, Jérôme Moreaux

**Affiliations:** 1 Institute of Human Genetics, UMR 9002 CNRS-UM, Montpellier, France; 2 CHU Montpellier, Laboratory for Monitoring Innovative Therapies, Department of Biological Hematology, Montpellier, France; 3 INSERM, U1040, Montpellier, France; 4 Cytometry IRB, Montpellier Rio Imaging, Montpellier, France; 5 CNRS UMR 7276, Université de Limoges, Limoges, France; 6 Pôle Cellules et Tissus, CHU Rennes, Rennes, France; 7 INSERM, U917, Rennes, France; 8 Université Montpellier 1, UFR Médecine, Montpellier, France; Institut Cochin, FRANCE

## Abstract

FCRL4 is an immunoregulatory receptor that belongs to the Fc receptor-like (FCRL) family. In healthy individuals, FCRL4 is specifically expressed by memory B cells (MBCs) localized in sub-epithelial regions of lymphoid tissues. Expansion of FCRL4^+^ B cells has been observed in blood and other tissues in various infectious and autoimmune disorders. Currently, the mechanisms involved in pathological FCRL4^+^ B cell generation are actively studied, but they remain elusive. As *in vivo* FCRL4^+^ cells are difficult to access and to isolate, here we developed a culture system to generate *in vitro* FCRL4^+^ B cells from purified MBCs upon stimulation with soluble CD40 ligand and/or CpG DNA to mimic T-cell dependent and/or T-cell independent activation, respectively. After 4 days of stimulation, FCRL4^+^ B cells represented 17% of all generated cells. Transcriptomic and phenotypic analyses of *in vitro* generated FCRL4^+^ cells demonstrated that they were closely related to FCRL4^+^ tonsillar MBCs. They strongly expressed inhibitory receptor genes, as observed in exhausted FCRL4^+^ MBCs from blood samples of HIV-infected individuals with high viremia. In agreement, cell cycle genes were significantly downregulated and the number of cell divisions was two-fold lower in *in vitro* generated FCRL4^+^ than FCRL4^-^ cells. Finally, due to their reduced proliferation and differentiation potential, FCRL4^+^ cells were less prone to differentiate into plasma cells, differently from FCRL4^-^ cells. Our *in vitro* model could be of major interest for studying the biology of normal and pathological FCRL4^+^ cells.

## Introduction

Memory B cells (MBCs) play a central role in immune memory thanks to their ability to rapidly differentiate into cells that secrete high affinity antibodies (Ab) in response to a secondary antigenic challenge. Several MBC subsets have been described, including a poorly characterized population of MBCs that express Fc receptor-like 4 (FCRL4) [1–3]. FCRL4 belongs to the family of Fc receptor-like proteins that are expressed mainly by the B-cell lineage [4]. Members of this family contain immunoreceptor tyrosine-based activation (ITAM) and/or inhibition motifs (ITIM) that could modulate their immunoregulatory potential. In healthy individuals, FCRL4^+^ B cells account for about 10% of tonsil B cells, whereas they are not detected in peripheral blood and bone marrow and are rare in the marginal zone of B cell follicles in spleen and lymph nodes [5–8]. Conversely, they are present in the peripheral blood of patients with chronic activation of the immune system, such as HIV-infected viremic individuals [9] and patients with hepatitis C virus-associated mixed cryoglobulinemia [10]. FCRL4^+^ B cells are also detected in the synovial fluid of patients with rheumatoid arthritis where they produce cartilage- and bone-destroying cytokines and could be the main target of anti-CD20 monoclonal antibody (mAb) therapy [11,12]. Moreover, FCRL4 polymorphisms have been associated with the susceptibility and severity of ankylosing spondylitis [13].

FCRL4^+^ MBCs are hyporesponsive to stimuli that activate classical MBCs and are considered to be exhausted MBCs [7,9]. Exhaustion of B-cell activation by HIV can be reversed by knocking down FCRL4 expression [14]. Also the HIV envelope protein gp120 binds to α4β7 integrin on B cells and induces TGFβ secretion and FCRL4 expression that are associated with B cell abortive proliferative response [15]. FCRL4 binds efficiently to IgA and could be important for immune complex-dependent B-cell regulation [16]. Compared with FCRL4^-^ MBCs, transcriptomic and proteomic analyses have shown that FCRL4^+^ B cells are characterized by overexpression of cell-surface molecules (particularly, CD20, CD11c and the chemokine receptors CCR1, CCR5 and CCR6), src-family kinases (*FGR*, *HCK*, *LYN*), transcription factors (*SOX5*, *RUNX2*, *DLL1)*, and activation-induced cytidine deaminase *(AICDA)* [6,7,9,10,12,17].

Given the link of FCRL4^+^ B cells with chronic diseases, it would be important to develop a culture model to better understand their generation and function. We previously reported that during differentiation of human MBCs into preplasmablasts (prePBs), we obtained a population of activated CD20^high^CD38^-^ B lymphocytes that strongly express *FCRL4* [18]. Here, we show that *in vitro* generated FCRL4^+^ B cells share multiple characteristics with *in vivo* FCRL4^+^ B cells. Differently from prePBs and PBs (FCRL4^-^ cells) generated using the same culture conditions, FCRL4^+^ cells have a low proliferation rate and are not prone to differentiate into plasmacells (PCs).

## Materials and methods

### Reagents

Human recombinant IL-2 was purchased from R&D Systems (Minneapolis, MN), IL-6 and IL-15 from AbCys SA (Paris, France), IL-10 from Peprotech (Rocky Hill, NJ), and IFN-α(Introna) from Merck Canada Inc. (Kirckland, Canada). Mouse mAbs conjugated to allophycocyanin (APC), fluorescein isothiocyanate (FITC), Pacific Blue (PB), peridinin chlorophyll protein-cyanin 5.5 (PerCP-Cy5.5), phycoerythrin (PE), BD Horizon Brilliant™ Blue 515 (BB515) against human CD1c (clone F10/21A3), CD19 (clone HIB19), CD22 (clone HIB22), CD24 (clone ML5), CD27 (clone M-T271), CD30 (clone BerH8), CD32 (clone FL18.26), CD38 (clone HIT2), CD40 (clone MAB89), CD72 (clone J4-117), CD196 (CCR6, clone 11A9), CD307c (FCRL3, clone H5) and immunoglobulin (Ig) -M (clone G20-127) were purchased from BD Biosciences (Le Pont De Claix, France); antibodies against CD11c (clone BU15), CD20 (clone B9E9), CD21 (clone BL13), CD126 (IL-6R, clone M91), and CD138 (clone B-A38) from Beckman Coulter (Fullerton, CA); antibodies against CD307d (FCRL4, clone H413D12) from Biolegend (San Diego, CA). Polyclonal goat Abs against human IgA and IgG were from Southern Biotech (Birmingham, AL).

#### Cell samples

Peripheral blood cells from healthy volunteers were purchased from the French Blood Center (EFS) Pyrénées-Méditérannée, Toulouse, France. After removal of CD2^+^ cells using anti-CD2 magnetic beads (Invitrogen, Cergy Pontoise, France), CD19^+^ CD27^+^ MBCs were sorted using a multi-color fluorescence FACS Aria device, as previously described [19]. When indicated, cells were FACS-sorted using a PE-conjugated anti-FCRL4 mAb. The purity of FACS-sorted cell populations was ≥95%, as assayed by cytometry.

### Cell cultures

**Step1. B-cell activation**. All cultures were performed in Iscove’s modified Dulbecco medium (IMDM, Invitrogen) and 10% fetal calf serum (FCS) (Invitrogen). Purified MBCs were cultured in the presence of 20 U/ml IL-2, 50 ng/ml IL-10 and 10 ng/ml IL-15 in 6-well culture plates (1.5 x 10^5^/ml cells in 5 ml/well). Ten μg/ml of phosphorothioate CpG ODN 2006 (ODN) (Sigma) [20], 50 ng/ml histidine-tagged recombinant human soluble CD40 ligand (sCD40L) and 5 μg/ml anti-poly-histidine mAb (R&D Systems) were added at the culture start. Step 2. PB generation. At day (D) 4 of culture, cells were harvested, washed and 2.5 x 10^5^/ml cells were seeded in IMDM/10% FCS with 20 U/ml IL-2, 50 ng/ml IL-6, 50 ng/ml IL-10 and 10 ng/ml IL-15. Step 3. PC generation. At D7 of culture, cells were harvested and 5 x 10^5^/ml cells were seeded in IMDM/10% FCS with 50 ng/ml IL-6, 10 ng/ml IL-15 and 500 U/ml IFN-α for 3 days.

### Immunophenotypic analysis

Cells were stained with combinations of mAbs conjugated to different fluorochromes. For intracellular staining of cytoplasmic (cy) IgM, -IgA, and–IgG, surface staining was performed prior to cell fixation and permeabilization using the Cytofix/Cytoperm kit (BD Biosciences), according to the manufacturer’s recommendations. Flow cytometry analysis was performed with a FACSAria cytometer using FACSDiva 6.1 (Becton Dickinson, San Jose, CA) and with a Cyan ADP cytometer driven by the Summit software (Beckman Coulter). For data analysis, Cell Quest (Becton Dickinson) and Summit, Kaluza (Beckman Coulter) softwares were used. The fluorescence intensity of the cell populations was quantified using the stain index (SI) formula: [mean fluorescence intensity (MFI) obtained from a given mAb minus MFI obtained with the control mAb]/[2 times the standard deviation of the MFI obtained with the same control mAb] [21].

### Analysis of Ig secretion

Flow cytometry sorted D4 FCRL4^+^ and FCRL4^-^ cells were cultured at 10^6^ cells/ml for 24 hours and culture supernatants harvested. IgM, IgA, or IgG concentrations were assessed by ELISA using human IgM, IgA, and IgG ELISA kits from Bethyl Laboratories (Montgomery, TX), according to the manufacturer's recommendations.

### CFSE labeling

Cell division was assessed by carboxyfluorescein succinimidyl ester (CFSE) labeling, as previously described [22]. Briefly, purified MBCs were washed and re-suspended at a concentration of 10^6^ cells/ml in PBS/0.1% BSA with 10 μM CFSE (Molecular Probes, Eugene, OR), incubated at 37°C for 10 minutes and extensively washed before culture. At D4 of culture, cells were washed and labeled with anti-PB-CD20, anti-PerCP-Cy5.5-CD38 and anti-FCRL4-PE mAbs for flow cytometry analysis. Cell divisions were quantified using the ModFit LT software (Verity Software House, Topsham, ME).

### Microarray hybridization and bioinformatic analysis

Cell RNA was extracted and hybridized to GeneChip® human genome U133 Plus 2.0 microarrays, according to the manufacturer’s instructions (Affymetrix, Santa Clara, CA). Gene expression data from D4 FCRL4^+^ and D4 FCRL4^-^ cells have been deposited in the Gene Expression Omnibus database (http://www.ncbi.nlm.nih.gov/geo/query/acc.cgi?acc=GSE87384; accession number GSE87384) and data from peripheral blood MBCs in the ArrayExpress public database (http://www.ebi.ac.uk/microarray-as/ae/); accession number E-MEXP-2360). Gene expression data were analyzed with our bioinformatics platform GenomicScape (www.genomicscape.com) [23]. Clustering was performed and visualized with Cluster and TreeView [24]. Differentially expressed genes between cell populations were identified with the significance analysis of microarray (SAM) statistical method (paired Wilcoxon statistics, fold change ≥ 2, 200 permutations, false decovery rate ≤ 0.01) [25,26]. Gene annotation and networks were generated with the Reactome Functional Interaction Cytoscape plugin (http://www.cytoscape.org/).

### Statistical analysis

Results were compared using the non-parametric Mann-Whitney test, unpaired or paired Student’s *t-*tests and the SPSS software. *P*-values ≤ .05 were considered significant.

## Results

### Generation of FCRL4^+^ B cells *In vitro*

**Fig 1:**
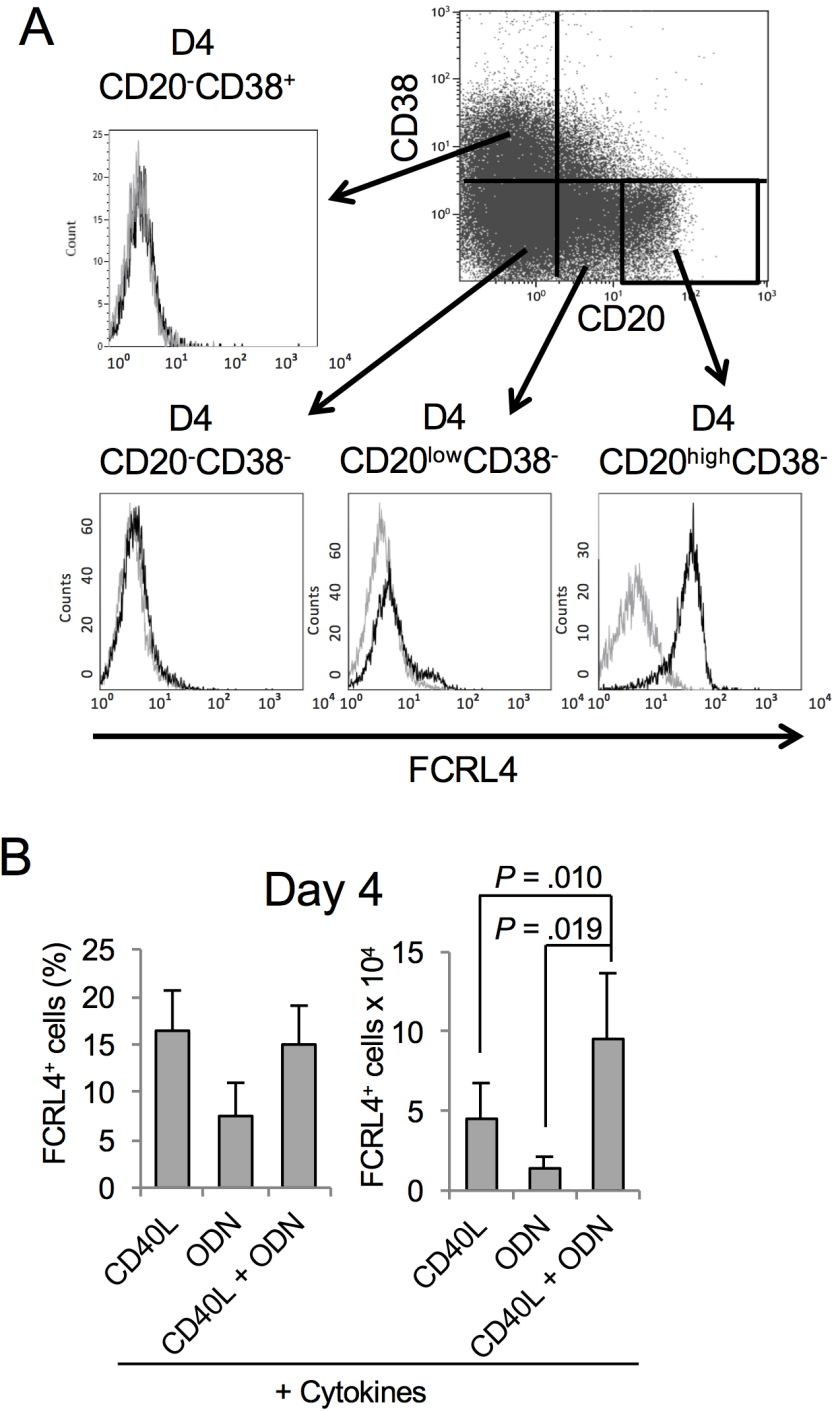
Activation of MBCs for 4 days leads to the generation of CD20^high^CD38^-^FCRL4^+^ cells. (**A**) MBCs were purified and cultured for 4 days in the presence of sCD40L, ODN and a cytokine cocktail (IL-2, IL-10 and IL-15). At day 4 (D4), expression of CD20, CD38 and FCRL4 was assessed by flow cytometry (representative results of one of 15 experiments). (**B**) MBCs were activated with sCD40L or ODN, or both, and the cytokine cocktail. At D4, FCRL4 expression was assessed by flow cytometry and cells counted by using the trypan blue exclusion method (representative results of four experiments).

Using our multi-step *in vitro* model of PC differentiation [18,19,27], we previously identified a population of activated CD20^high^ B lymphocytes generated after 4 days of stimulation by sCD40L and/or ODN and a cytokine cocktail (IL-2, IL-10 and IL-15). Activated CD20^high^ B cells represented 17% of all D4 cells that were mainly CD20^low/-^CD38^-^ prePBs and CD20^-^CD38^+^ PBs. Microarray-based gene expression profiling of these cells showed that 267 genes were overexpressed in purified CD20^high^ B cells compared with prePBs (fold change ≥2; false discovery rate <0.05). Among these genes, *FCRL4* was the second most overexpressed gene [18]. Flow cytometry analysis confirmed FCRL4 overexpression in CD20^high^ B cells compared with prePBs or PBs (Fig 1A). FCRL4^+^ B cells were generated after MBC activation with sCD40L or ODN that mimicks, respectively, T-cell dependent and T-cell independent stimulation. Their combination did not increase the FCRL4^+^/FCRL4^-^ cell ratio, but the total number of generated (FCRL4^+^ and also FCRL4^-^) cells was significantly enhanced (Fig 1B, *P <0*.*02*).

### Characterization of FCRL4^+^ B cells

**Fig 2:**
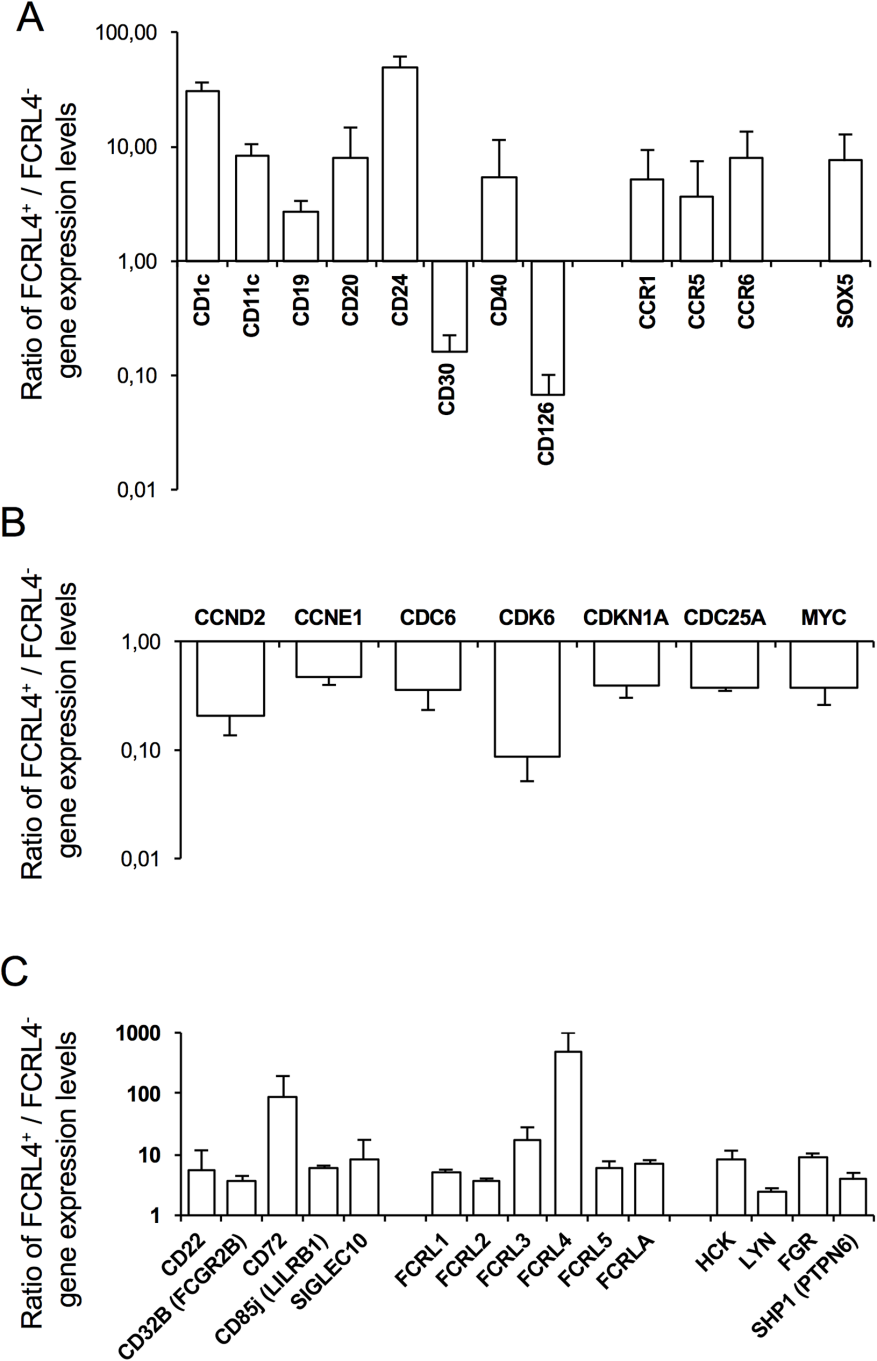
Genes differentially expressed by FCRL4^+^ and FCRL4^-^ cells at D4 of culture. MBCs from three healthy donors were activated with sCD40L and ODN with cytokines for 4 days. At D4, FCRL4^+^ and FCRL4^-^ cells were cell-sorted by flow cytometry and RNA was extracted and hybridized to GeneChip® human genome U133 Plus 2.0 microarrays. Genes differentially expressed in the two cell populations were identified using the SAM statistical method (*P* < 0.05; fold change ≥ 2 and false decovery rate ≤ 0.01). (**A**) Genes encoding cell surface markers, chemokine receptors and transcription factors. (**B**) Genes encoding cell cycle proteins. (**C**) Genes encoding ITIM-containing receptors and downstream signaling molecules.

**Fig 3:**
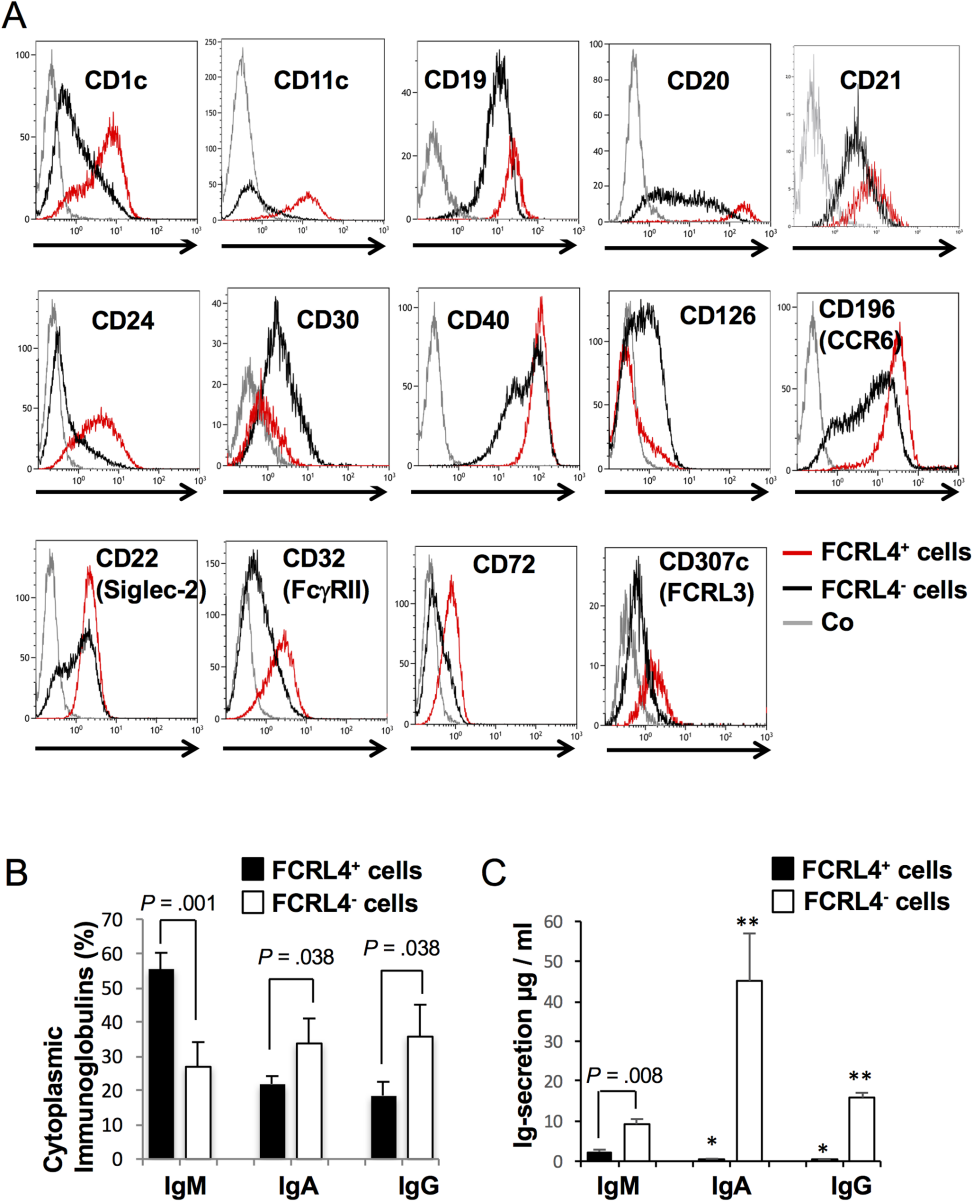
Differential expression of cell surface markers and IgH isotypes in D4 FCRL4^+^ and FCRL4^-^ cells. **(A)** Differential expression of cell surface markers in FCRL4^+^ and FCRL4^-^ cells at D4 of culture. MBCs were activated as indicated in Fig 2. At D4 of culture, FCRL4^+^ (red) and FCRL4^-^ (black) cells were labeled with mAbs against various surface markers and against FCRL4 and their expression analyzed by flow cytometry. Gray, negative control antibodies (Co). Results are representative of three or more experiments. **(B)** Expression of IgH isotypes in FCRL4^+^ and FCRL4^-^ cells at D4 of culture. Cells were first labeled with an anti-FCRL4 mAb, then fixed, permeabilized and labeled with anti-IgM, anti-IgA and anti-IgG Abs. Results are the mean percentage of positive cells ± SD for each isotype of four separate experiments. **(C)** IgM, IgA and IgG secretion by D4 FACS-sorted FCRL4^+^ and FCRL4^-^ cells was assessed by ELISA. Results are the mean ± SD of Ig concentration in μg per ml of 24 hours culture supernatants determined in 3 separate experiments. * IgA and IgG secretion are significantly weaker than IgM secretion in D4 FCRL4^+^ cells (*P* ≤ 0.027). ** IgA and IgG secretion are significantly higher than IgM secretion in D4 FCRL4^-^ cells (*P* ≤ 0.027).

To specifically characterize FCRL4^+^ B cells, whole genome transcriptome analysis was performed using purified *in vitro* generated FCRL4^+^ and FCRL4^-^ B cells at D4 (three independent experiments). Paired SAM supervised analysis (ratio ≥2, false discovery rate ≤0.01) identified 527 genes that were significantly upregulated and 457 genes that were downregulated in FCRL4^+^ compared with FCRL4^-^ cells (S1 Table). *In vitro*-derived FCRL4^+^ cells displayed a gene expression profile close to that of *ex vivo* FCRL4^+^ B cells [7,17], with high expression of genes encoding various surface markers, including CD20, CD11c and CD40, several members of the chemokine receptor family (CCR1, CCR5, and CCR6) and the SOX5 transcription factor (Fig 2A). Flow cytometry analysis confirmed overexpression of CD11c, CD20, CD40 and CCR6 (CD196) and of other surface proteins, including CD1c, CD19 and CD24, in FCRL4^+^ cells (Fig 3A, Table 1). Downregulation of CD30 (TNFRSF8) and CD126 (IL6R) in FCRL4^+^ compared with FCRL4^-^ B cells was also validated by flow cytometry (Fig 3A, Table 1). CD21 expression, *in vitro*, is higher in FCRL4^+^ cells compared to FCRL4^-^ cells at D4. Same results were obtained at D1 (data not shown). These data contrast to the low CD21 expression reported on FCRL4^+^ cells *ex vivo* [6,7,9,10,12,17,28,29] and could be a feature of *in vitro* generated FCRL4^+^ cells.

**Table 1:** Differential expression of cell surface markers in FCRL4^+^ and FCRL4^-^ cells at D4 of culture.

	Positive cells %		*P*	Stain Index		*P*
	FCRL4^+^ cells	FCRL4^-^ cells		FCRL4^+^ cells	FCRL4^-^ cells	
CD1c	76.5 ± 8.7	28.2 ± 8.0	0.005	16.4 ± 6.8	3.4 ± 1.7	0.04
CD11c	74.6 ± 13.0	15.8 ± 0.8	0.002	14.4 ± 7.9	1.7 ± 0.6	0.03
CD19	99.9 ± 0.1	98.0 ± 1.4	ns*	89.1 ± 16.0	42.6 ± 9.8	0.006
CD20	100 ± 0.1	34.8 ± 5.3	0.000001	368 ± 183	44.3 ± 19.1	0.004
CD21	98.3 ± 0.3	76.8 ± 3.5	0.006	22.8 ± 5.4	9.0 ±1.4	0.01
CD24	64.7 ± 10.7	4.7 ± 6.7	0.00006	11.0 ± 7.4	0.7 ± 1.2	0.010
CD30	48.4 ± 15.7	77.1 ± 13.9	0.00007	4.8 ± 1.3	16.5 ± 5.1	0.003
CD40	99.9 ± 0.1	99.4 ± 0.7	ns	165 ± 59	112 ± 44	0.02
CD126	14.0 ± 4.0	42.1 ± 14.6	0.010	0.8 ± 0.3	3.0 ± 1.5	0.02
CD196	99.0 ± 0.6	79.6 ± 3.7	ns	90.3 ± 25.1	39.2 ± 8.3	0.02
CD22	98.4 ± 1.0	68.0 ± 8.7	0.015	9.9 ± 0.7	5.9 ± 0.7	0.0003
CD32	86.5 ± 4.9	43.3 ± 14.1	0.012	13.3 ± 4.9	5.6 ± 3.4	0.01
CD72	30.6 ± 14.4	7.0 ± 4.0	0.04	1.9 ± 0.5	0.6 ± 0.3	0.007
CD307c	75.3 ± 6.1	43.9 ± 8.9	0.04	17.9 ± 14.2	7.0 ± 4.6	ns

*ns: not significantly different

Moreover, pathway enrichment analysis using Reactome (false discovery rate <0.001) showed that genes coding for proteins involved in B cell activation, B cell receptor signaling, antigen processing and presentation were significantly enriched in FCRL4^+^ cells (S2 Table). Conversely, genes involved in cell cycle and cell proliferation were significantly downregulated in FCRL4^+^ B cells compared with FCRL4^-^ cells (Fig 2B and S2 Table).

*In vitro* generated FCRL4^+^ cells expressed the same inhibitory receptor genes than the FCRL4^+^ tissue-like memory B cells described by Moir and collaborators in blood of HIV-positive viremic individuals [9,14]. Particularly, the genes encoding CD22 (Siglec-2), CD32B (FcγRIIB), CD72 and CD85j (LILRB1) (Fig 2C), which have an ITIM intracytoplasmic domain and inhibit B-cell receptor (BCR) signaling, were overexpressed in FCRL4^+^ cells. In addition, other ITIM receptor genes, such as *Siglec-10* and the FCRL family members *FCRL1*, *FCRL2*, *FCRL3 (CD307c)* and *FCRL5*, were upregulated in FCRL4^+^ compared with FCRL4^-^ B cells (Fig 2C). CD22, CD32, CD72 and FCRL3 overexpression in FCRL4^+^ B cells was validated by flow cytometry analysis (Fig 3A, Table 1). Genes encoding the src-tyrosine kinase family members HCK, FGR, LYN and the tyrosine phosphatase SHP1 were also upregulated in FCRL4^+^ cells (Fig 2C). These tyrosine kinases have been shown to phosphorylate ITIM domains enabling the recruitment of tyrosine phosphatases SHP1 and SHP2 [25,30].

Differently from what previously reported [17], cell cycle modulators and *AICDA* were not overexpressed in D4 FCRL4^+^ cells compared with D4 FCRL4^-^ cells. This study reported the gene expression profile of FCRL4^+^ and FCRL4^-^ MBCs purified from human tonsils, whereas in the present work *in vitro* activated FCRL4^+^ B cells and highly proliferating FCRL4^-^ prePB/PB cells were investigated. Therefore, to identify genes that are overexpressed in both tonsils FCRL4^+^ MBCs and in *in vitro*-generated FCRL4^+^ B cells, D4 FCRL4^+^ B cells and MBCs (which are FCRL4^-^) purified from blood samples (D0 cells) [19] were compared. This should allow identifying additional genes that are upregulated in FCRL4^+^ cells, but that could not be detected in the first transcriptome analysis because expressed also by *in vitro*-generated D4 FCRL4^-^ cells. SAM supervised analysis (ratio ≥4, false discovery rate <0.005) identified 655 genes that were significantly upregulated in FCRL4^+^ B cells (S3 Table), including genes that are typically expressed in FCRL4^+^ cells (*CD1c*, *CD24*, *CCR1*, *CCR5*, *CCR6*, *CD72*, *HCK*, *FCRL5*). Among the 128 genes that were significantly overexpressed in FCRL4^+^ MBCs from tonsils [17], 61 (48% of 128 genes) were also overexpressed in *in vitro*-generated FCRL4^+^ B cells (S4 Table), including genes encoding cell cycle modulators and *AICDA*. This underlines the strong similarity of *in vivo* and *in vitro*-generated FCRL4^+^ B cells.

Finally, the expression of IgH isotypes was assessed in D4 FCRL4^+^ and FCRL4^-^ cells. As we previously showed for D4 CD20^high^CD38^-^ B cells [18], FCRL4^+^ cells expressed most frequently IgM than FCRL4^-^ cells. Conversely, class switching to IgA or IgG was about 2-fold less frequent in FCRL4^+^ than in FCRL4^-^ cells (Fig 3B). Moreover, the staining intensity for each isotype was two-fold weaker in FCRL4^+^ B cells (ratio of 2.5 ± 0.1 for IgM; 2.4 ± 0.6 for IgA and 2.4 ± 0.3 for IgG). Analysis of Ig secretion by ELISA (Fig 3C) confirmed flow cytometry results. Cell-sorted D4 FCRL4^+^ cells produce mainly IgM (ratio IgM/IgA+IgG = 4.1 ± 1.6). Conversely, FCRL4^-^ cells produce mainly class-switched Igs (ratio IgA/IgM = 5.0 ± 1.8 and IgG/IgM = 1.7 ± 0.6). Of note, although FCRL4^+^ cells produce essentially IgM, this IgM production is significantly weaker when compared to FCRL4^-^ cells. This is in agreement with the cytoplasmic IgM stain index. General characteristics of in vitro generated FCRL4^+^ cells and primary FCRL4^+^ cells isolated from tonsils or tissues in disease-states are summarized in S5 Table.

### Mechanisms of FCRL4^+^ B-cell generation

**Fig 4:**
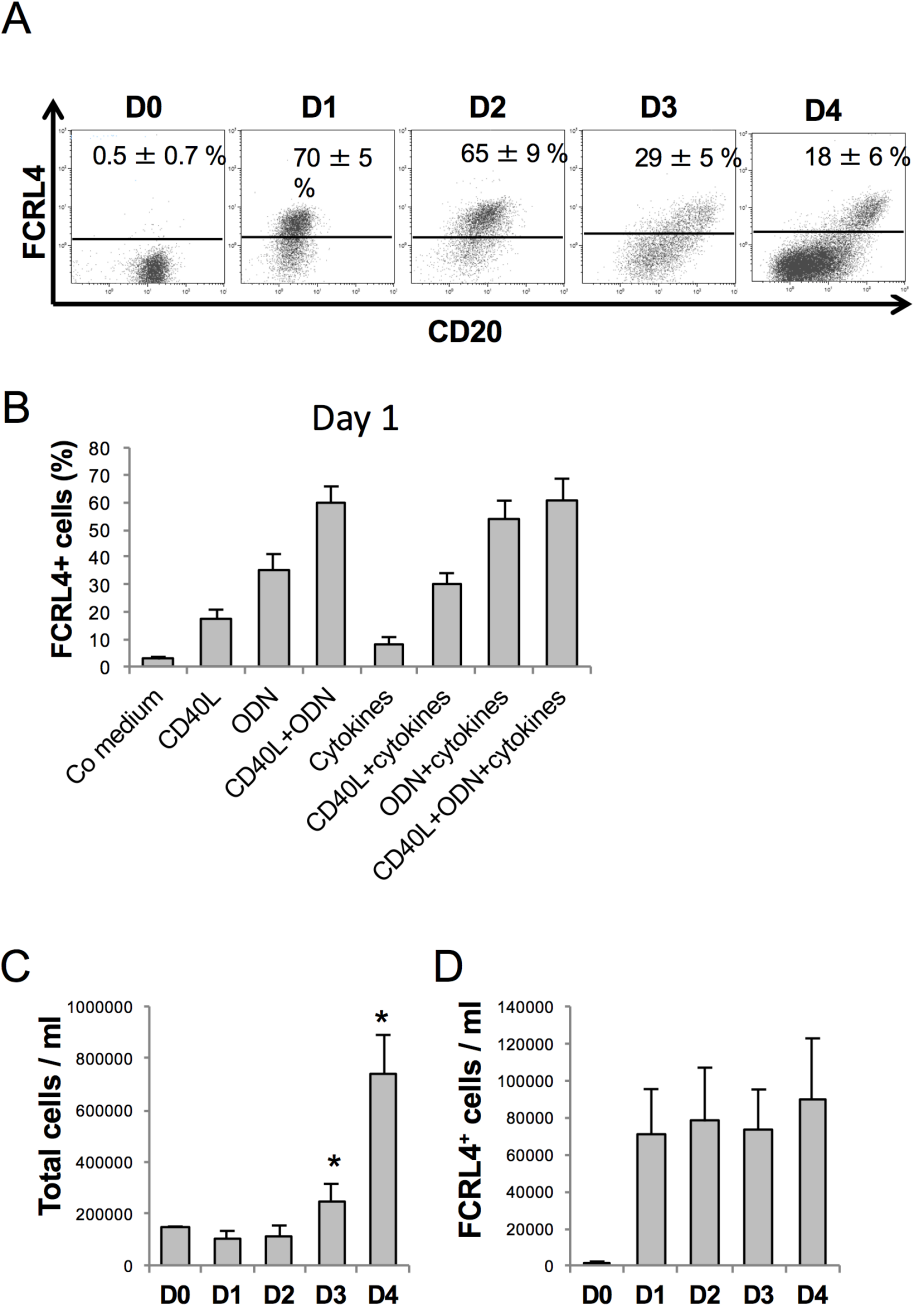
Kinetic analysis of FCRL4 expression. (**A**) MBCs were activated with CD40L+ODN with cytokines. Cells were harvested at the different time points and labeled with anti-FCRL4 and anti-CD20 mAbs. The mean percentage ± SD of FCRL4^+^ cells (n = 6 experiments) was determined by flow cytometry and is indicated in each plot. (**B**) MBCs were activated with control (Co) medium, or CD40L, ODN, CD40L+ODN with or without cytokines. At D1 of culture cells were labeled with an anti-FCRL4 mAb and the mean percentage ± SD of FCRL4^+^ cells (n = 3 experiments) was analyzed by flow cytometry. (**C**) and (**D**) MBCs were activated with CD40L, ODN or CD40L+ODN with cytokines. The total cell number was calculated using the trypan blue exclusion method and the percentage of FCRL4^+^ cells by flow cytometry to determine the number of FCRL4^+^ cells from D0 to D4 of culture. Results are the mean ± SD of six experiments. * *P* < .02 compared with D0.

**Fig 5:**
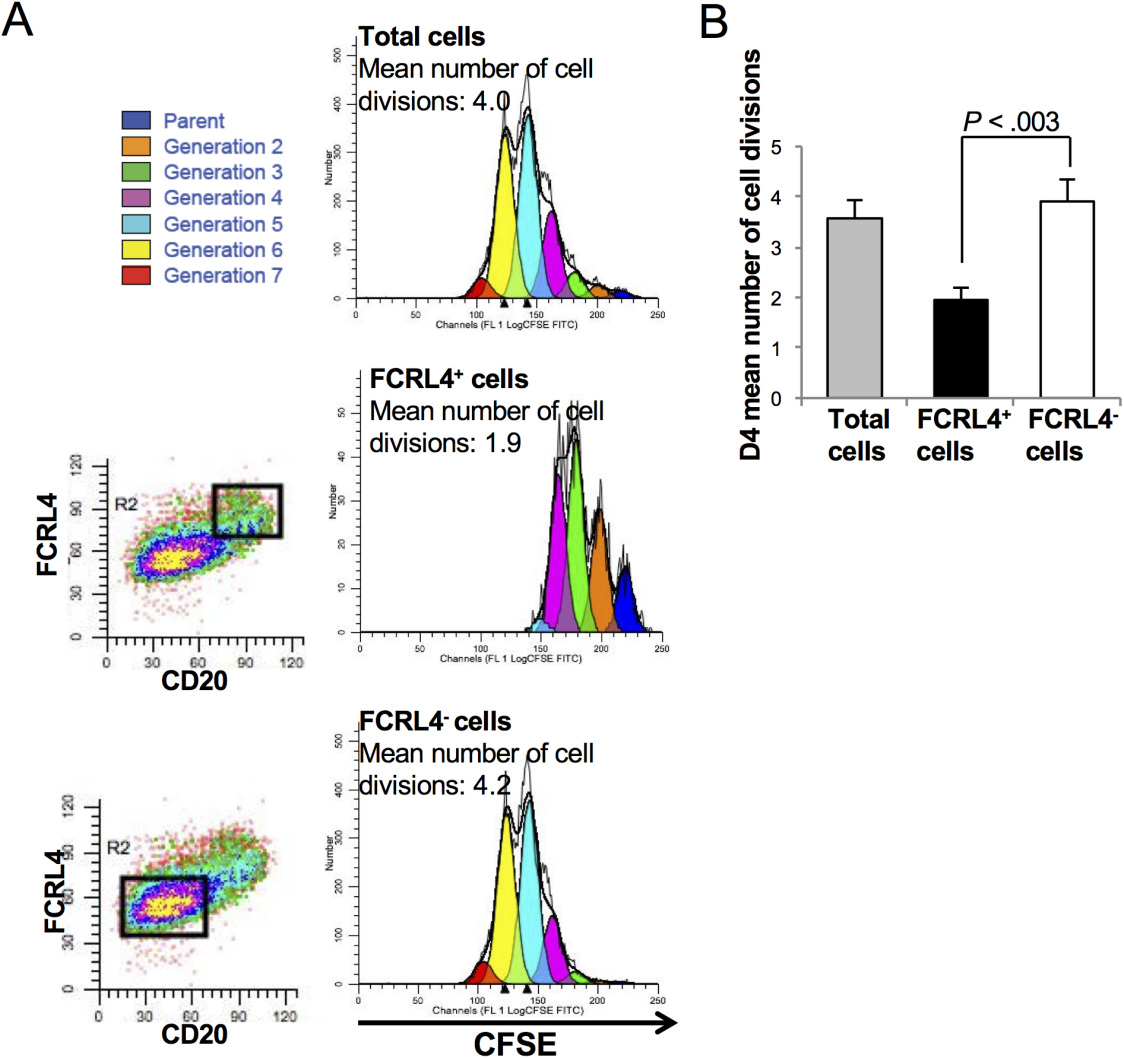
Significant decreased cell divisions in D4 FCRL4^+^ cells compared to D4 FCRL4^-^ cells. MBCs were labeled with CFSE at D0 and activated with CD40L+ODN with cytokines. The decrease of CFSE signal, due to cell division, was evaluated at D4 by flow cytometry in total cells and by gating on FCRL4^+^ and FCRL4^-^ cells. Results were processed with the ModFit Lt software to determine the number of cell divisions. (**A**) Representative experiment. The mean number of cell divisions is indicated. (**B**) Results are the mean ± SD of five separate experiments.

**Fig 6:**
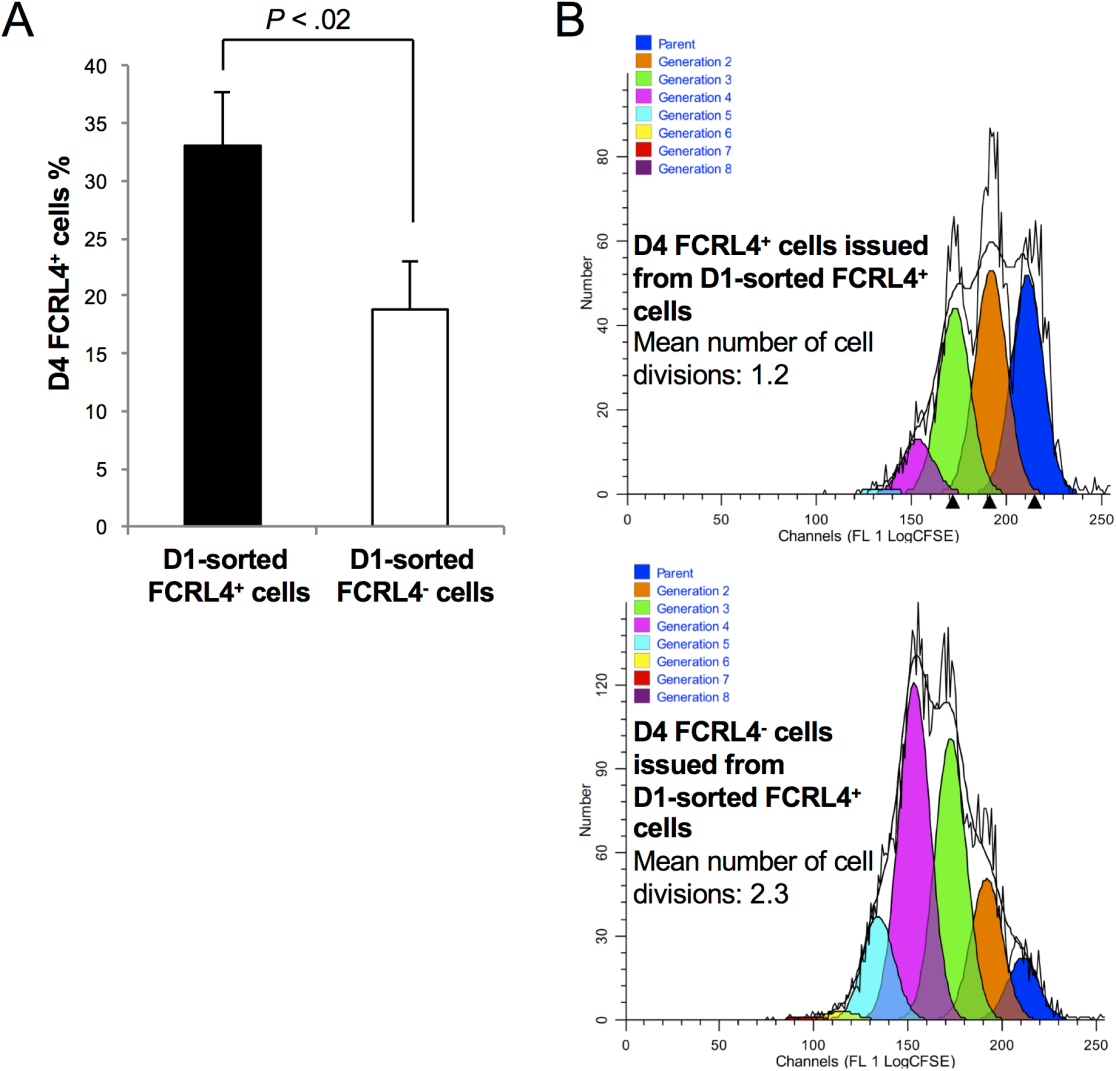
Only a minority of D1-sorted FCRL4^+^ cells maintains FCRL4 expression at D4 of culture and D4-FCRL4^+^ have a decreased mean number of cell divisions. MBCs were activated as indicated in Fig 2, labeled with an anti-FCRL4 mAb at D1 of culture to sort FCRL4^+^ and FCRL4^-^ cells by flow cytometry and to culture them separately. **(A)** At D4 of culture, FCRL4 expression was assayed again by flow cytometry. Results are the mean ± SD of four separate experiments. **(B)** D1-sorted FCRL4^+^ cells were labeled with CFSE and the decrease of CFSE signal, due to cell division, was analyzed at D4 in FCRL4^+^ and FCRL4^-^ cells by flow cytometry. Results were processed with the ModFit Lt software to determine the number of cell divisions. Results are representative of one experiment out of two. The mean number of cell divisions is indicated.

Kinetic analysis of FCRL4 expression indicated that it was induced in 70% of CD20^+^ B cells one day (D1) after activation with CD40L, ODN, IL-2, IL-10 and IL-15 (Fig 4A). At this time, cells had not proliferated and activation by ODN and/or CD40L was required for induction of FCRL4 expression (Fig 4B). Cytokines alone induced FCRL4 expression only in few cells, and addition of the cytokine cocktail did not increase the percentage of FCRL4^+^ cells at D1 compared with CD40L and/or ODN alone (Fig 4B). The percentage of FCRL4^+^ cells decreased by two-fold at D3 and by four-fold at D4, although the total number of cells increased significantly between D3 and D4 due to proliferation (Fig 4C). Indeed, the absolute number of FCRL4^+^ cells did not increase from D1 to D4 (Fig 4D). Several hypotheses could explain this observation. FCRL4^+^ B cells might proliferate less vigorously from D1 to D4 than FCRL4^-^ cells. A fraction of FCRL4^+^ B cells might also differentiate into FCRL4^-^ prePBs and/or die of apoptosis. This last hypothesis was unlikely because FCRL4+ cell viability was stable from D1 to D4. On the other hand, evaluation of FCRL4^+^ B cell proliferation by CFSE labeling at D0 followed by fluorescence measurement at D4 showed that the mean number of cell divisions in D4 FCRL4^+^ cells was 2-fold lower than in FCRL4^-^ cells (1.95 versus 3.91 respectively) (Fig 5A and 5B). These results were consistent with the downregulation of cell cycle genes and upregulation of inhibitory receptor genes in FCRL4^+^ B cells (Fig 2B and 2C). Their reduced proliferation rate might explain the progressive reduction of the percentage of FCRL4^+^ cells from D1 (70%) to D4 (18%). To determine whether some D1 FCRL4^+^ cells differentiated into FCRL4^-^ cells, FCRL4^+^ and FCRL4^-^ B cells were sorted at D1 and cultured separately. Only 32% of sorted FCRL4^+^ cells retained FCRL4 expression at D4 (Fig 6A). Conversely, most (82%) purified FCRL4^-^ cells remained FCRL4 negative. As shown in Fig 5 with unsorted cells, CFSE labeling experiments on D1-sorted FCRL4^+^ cells were done and confirmed that at D4, FCRL4^+^ cells had a two-fold decrease of their mean number of cell divisions compared to FCRL4^-^ cells (1.2 ± 0.1 versus 2.3 ± 0.1 respectively, Fig 6B). Altogether, these results indicate that FCRL4 expression at the beginning of the culture is mostly acquired by a sub-population of cells (70%) with lower proliferation rate. Moreover, these cells can further differentiate into FCRL4^-^ cells. This explains the progressive decrease of the FCRL4^+^/FCRL4^-^ cell ratio from D1 to D4.

### Plasma cell differentiation potential of FCRL4^+^ and FCRL4^-^ cells

**Fig 7:**
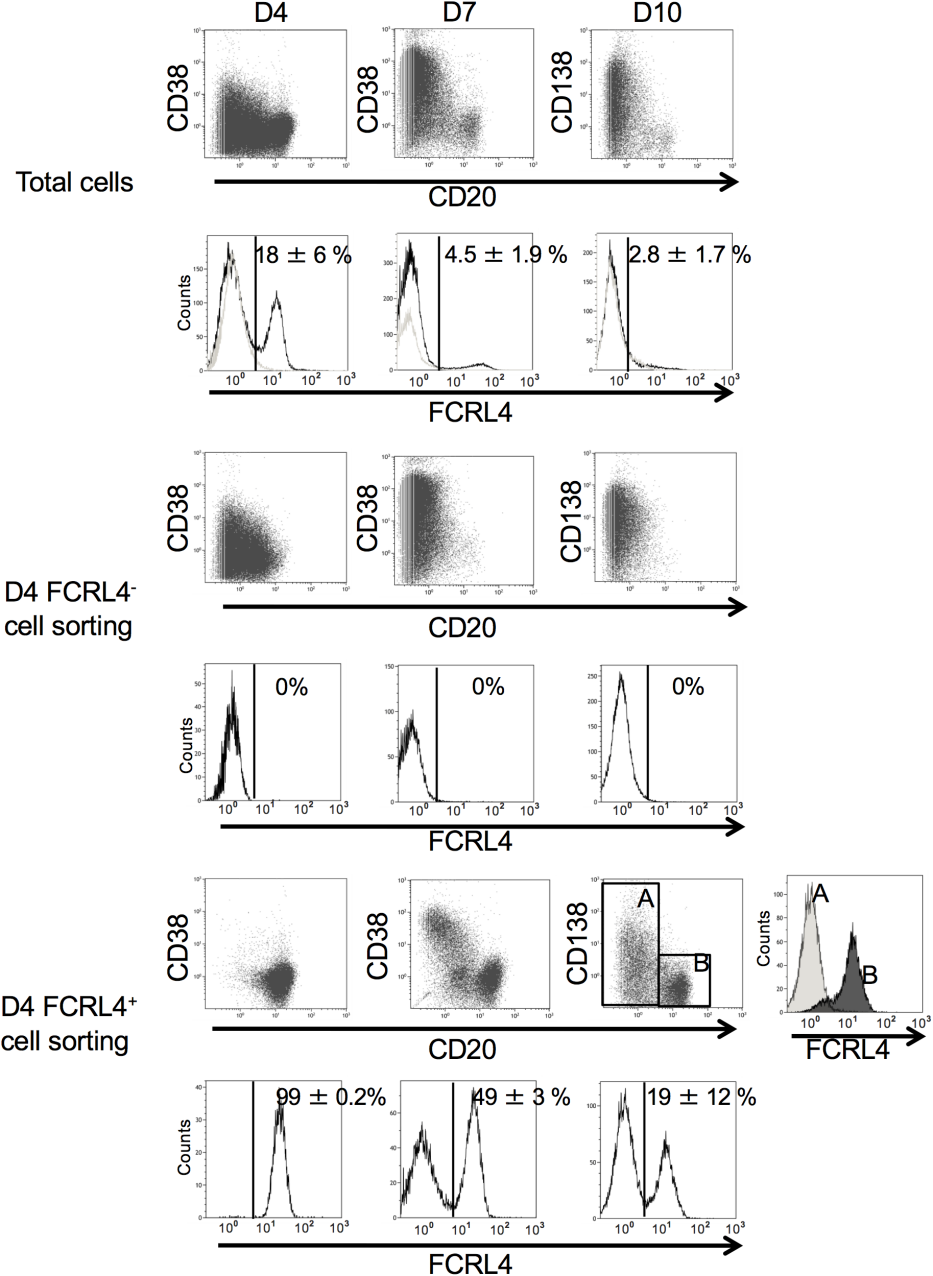
Plasma cell differentiation potential of FCRL4^+^ cells. MBCs were activated as indicated in Fig 2, labeled with an anti-FCRL4 mAb at D4 of culture and FCRL4^+^ and FCRL4^-^ cells were sorted by flow cytometry and cultured separately. At D7 and D10 of culture, expression of CD20, CD38, CD138 and FCRL4 was analyzed in non-sorted cells (total cells) and sorted FCRL4^+^ and FCRL4^-^ cells by flow cytometry. FCRL4 expression in PBs/PCs (CD20^-^ CD138^-^/CD20^-^ CD138^+^) and B cells (CD20^+^) generated from sorted D4 FCRL4^+^ cells is shown (windows A and B, respectively). Results are representative of six experiments.

**Fig 8:**
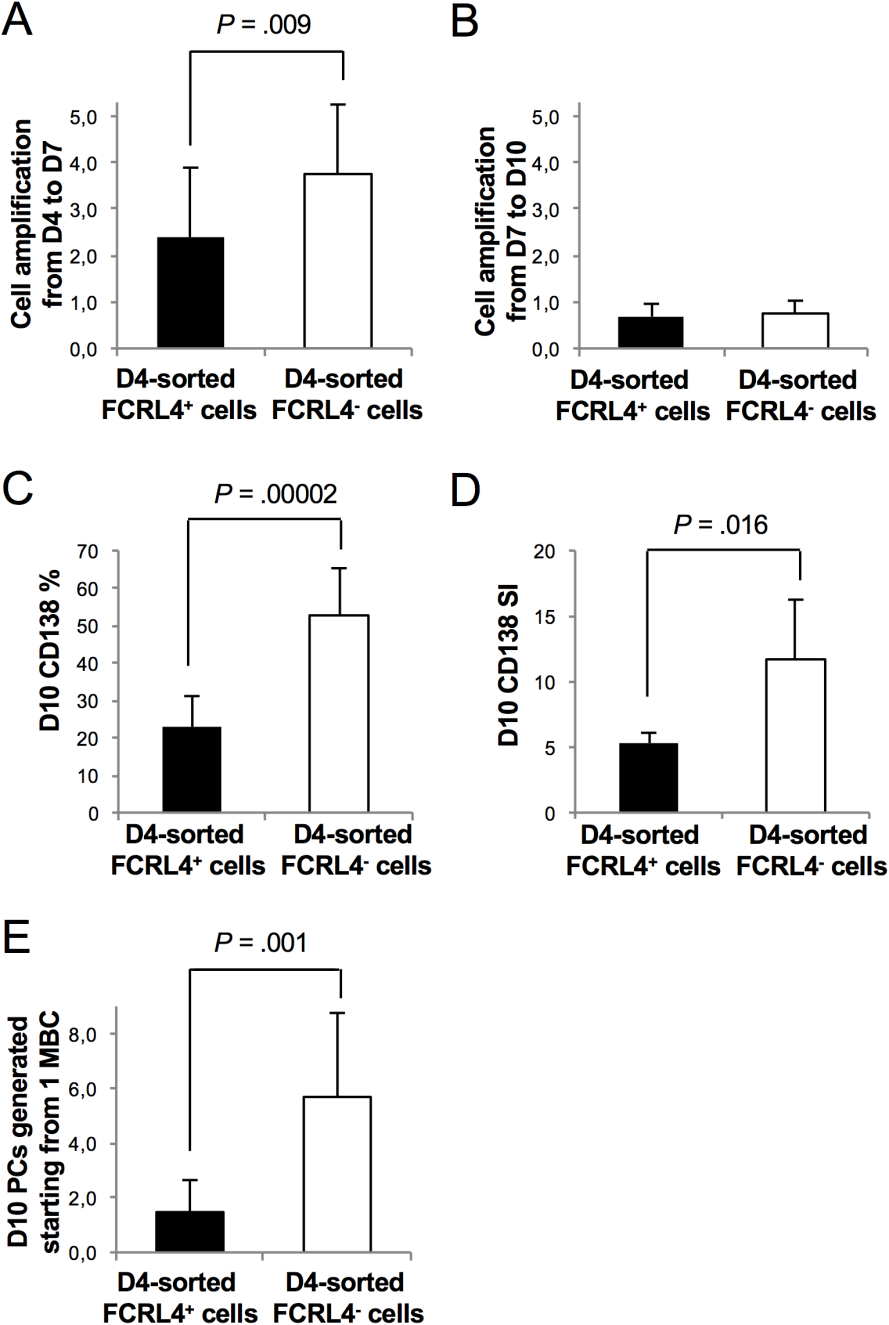
FCRL4^+^ cells demonstrate low expansion property and generate a reduced number of PCs. As in Fig 7, FCRL4^+^ and FCRL4^-^ cells were sorted at D4 and cultured separately until D7 and D10. (**A**) Total (unsorted) cells were counted at D7 and cell amplification determined as the ratio of total D7 cell count/number of cells seeded at D4 (results are the mean ± SD of 10 experiments). (**B**) Total cells were counted at D10 and cell amplification determined as the ratio of total D10 cell count/number of cells seeded at D7 (results are the mean ± SD of 10 experiments). (**C**) Percentage of CD138^+^ PCS at D10 (mean ± SD of 9 experiments). (**D**) Stain index (SI) of CD138 at D10 (mean ± SD of 5 experiments). (**E**) Number of PCs generated starting from 1 MBCs (mean ± SD of 9 experiments).

To compare the PC differentiation potential of FCRL4^+^ and FCRL4^-^ cells *in vitro*, D4 cells (end of step 1, see Materials and Methods) were purified on the basis of FCRL4 expression and cultured in conditions that promote PB differentiation for 3 days (step 2) and then in conditions that favor PC differentiation for another 3 days (step 3) (Fig 7) [18,19,27]. During step 2 (D4 to D7), FCRL4^-^ cells expanded 3.8-fold and differentiated into CD38^high^CD20^-^ PBs and then, during step 3 (from D7 to D10), into CD138^+^CD20^-^ PCs without expansion (Figs 7, 8A and 8B). This is not surprising because we previously found that D4 CD20^-/low^CD38^-^ cells and D4 CD20^-^CD38^+^ cells (representing D4 FCRL4^-^ cells) are prePBs and PBs already committed to PC differentiation [18]. On the other hand, fewer D4-sorted FCRL4^+^ cells differentiated into PBs and cell amplification was less important (step 2) compared with D4 FCRL4^-^ cells (Figs 7 and 8A). At D10, about 19% of D4-sorted FCRL4^+^ cells still expressed FCRL4 and had not differentiated into PBs and PCs, as indicated by the absence of CD38 and maintenance of CD20 expression (Fig 7). The other FCRL4^+^ cells differentiated into CD20^-^CD38^high^ FCRL4^-^ PBs and then into CD138^+^ PCs. However, at D10, the percentage of CD138^+^ cells and the level of CD138 expression (stain index) were significantly lower (2.3-fold and 2.2-fold, respectively) in D4-sorted FCRL4^+^ than in D4-sorted FCRL4^-^ cells (Fig 8C and 8D). Indeed, due to their reduced proliferation and differentiation potential, at D10, the number of PCs derived from D4-purified FCRL4^+^ B cells was four times lower compared with that from sorted FCRL4^-^ B cells (Fig 8E). In agreement with the class switching status of D4 cells, the ratio of IgM-expressing cells was 2.1-fold higher in D10 PCs derived from D4-sorted FCRL4^+^ B cells than from D4-sorted FCRL4^-^ cells (S1 Fig). The limited number of D10 PCs derived from D4 FCRL4^+^ cells did not allowed to sort them and measure Ig secretion.

The same analysis was performed by purifying FCRL4^+^ and FCRL4^-^ as soon as D1 of culture and assessing cell phenotype and cytoplasmic Igs expression at each step of PC differentiation (S2 Fig). We observed a rapid decrease of D4 FCRL4^+^ cells generated from D1-sorted FCRL4^+^ and D1-sorted FCRL4^-^ cells at the end of step 2 (D7) and step 3 (D10, FCRL4^+^ cells < 1%) (S2A Fig). D1-sorted FCRL4^-^ cells produced more prePBs (D4), PBs (D7) and PCs (D10) compared to D1-sorted FCRL4^+^ cells (S2B Fig). The percentage of cytoplasmic Igs in FCRL4^+^ and FCRL4^-^ cells derived from D1-sorted cells is indicated in S2C and S2D Fig. The ratio of IgM-expressing cells was again 2-fold higher in D10 PCs derived from D1-sorted FCRL4^+^ B cells compared to D10 PCs derived from D1-sorted FCRL4^-^ cells (S2E Fig), as observed when sorting the cells at D4 (S1 Fig).

Altogether, these results show that FCRL4^+^ cells are less prone to differentiate into PCs compared with FCRL4^-^ cells.

## Discussion

**Fig 9:**
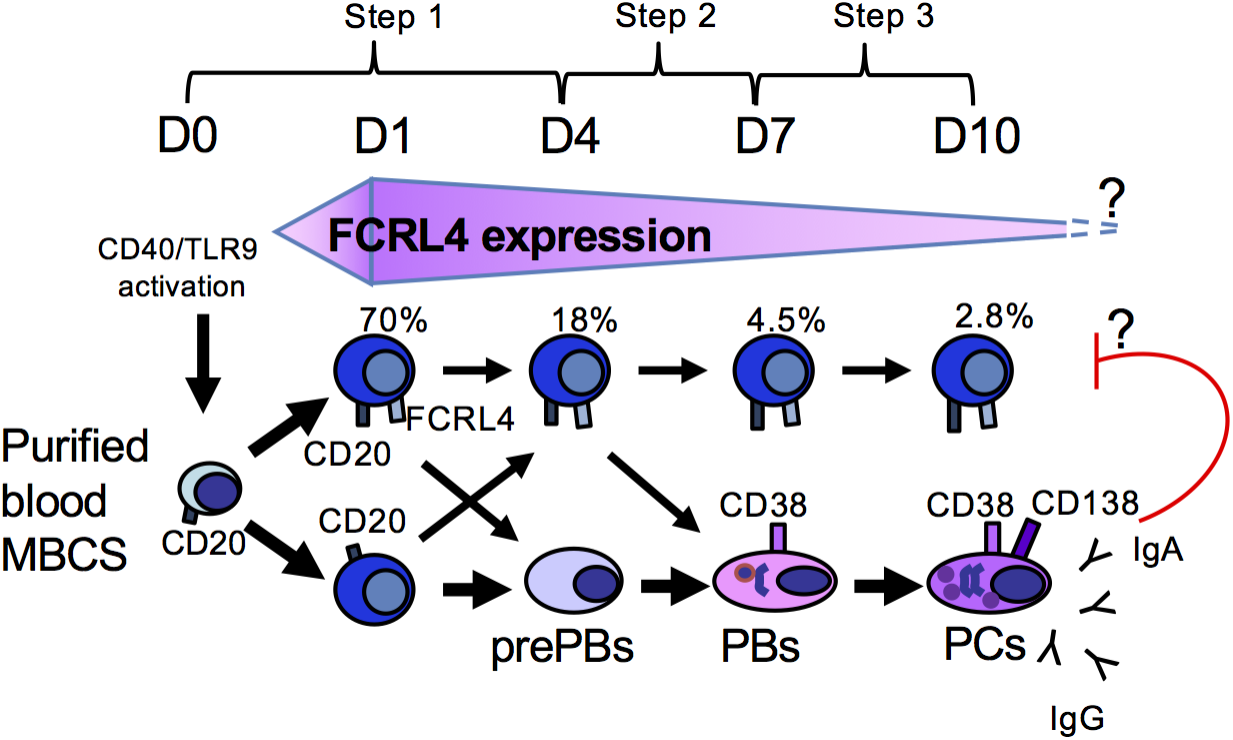
In vitro generation of FCRL4^+^ B cells. The majority of MBCs from blood express FCRL4 24 hours after stimulation. These cells can express FCRL4 transitorily and have a low proliferation. Indeed, only a few of cells remains positive at D10. FCRL4 is a receptor for IgA and IgA production by PBs and PCs could transduce inhibitory signals inhibiting FCRL4^+^ cell proliferation in our model.

The presence of atypical MBCs FCRL4^+^ in the blood has been described in different pathologies associated with chronic activation of the immune system [9]. In healthy individuals, the localization of FCRL4^+^ B cells is essentially limited to mucosa-associated lymphoid tissues (tonsils, Peyer’s patches) [5–8]. Here, using our *in vitro* PC differentiation model [19] we generated activated B cells that express FCRL4. Within four days after MBC activation by CD40L, ODN and a cytokine cocktail, two distinct populations are detected: activated FCRL4^+^ B cells and FCRL4^-^ prePBs. Transcriptomic and flow cytometry analyses of these cells demonstrated that the expression profile of *in vitro*-generated D4 FCRL4^+^ B cells is close to that of FCRL4^+^ cells observed in tonsils and in some pathologies. Particularly, 48% of the genes overexpressed by FCRL4^+^ MBCs in tonsils (compared with FCRL4^-^ MBCs in tonsils) [17] were also overexpressed in *in vitro*-generated FCRL4^+^ cells (compared with purified blood MBCs), despite their radically different environment (tonsils vs *in vitro* culture). FCRL4 expression appears mainly one day after CD40L stimulation and is detected in the majority of activated cells. Part of D1 FCRL4^+^ cells further mature into FCRL4^+^CD20^high^ B cells and the others into FCRL4^-^CD20^-/low^ prePBs. At D1, cells are viable and have not proliferated, indicating that most MBCs purified from blood have the potential to express FCRL4. This suggests that *in vitro*-generated FCRL4^+^ cells do not constitute a specific MBC class. According to these results, FCRL4 expression results from sustained MBC activation and can be transient (Fig 9).

This could explain why in healthy individuals, FCRL4^+^ MBCs are located in tonsils (i.e., a site particularly exposed to pathogens and with continuous ongoing immune responses). In the case of HIV-infected patients with high viremia or patients with rheumatoid arthritis, chronic immune activation could induce the generation of FCRL4^+^ B lymphocytes in additional sites where they are not usually present, including blood or synovial fluid. [9,10,12] The chemokine receptor profile (CCR1, CCR5 and CCR6 expression) of FCRL4^+^ cells suggests that they are prone to migrate to inflammatory tissues.

In agreement with the downregulation of cell cycle gene expression in FCRL4^+^ B cells, their proliferation is two-fold lower compared with prePBs. This could explain the low proportion of FCRL4^+^ cells at D4. The low proliferation rate of FCRL4^+^ B cells is consistent with the inhibitory activity of this receptor on BCR signaling [7,25,31] and its ability to induce B cell exhaustion in HIV-infected individuals with high viremia and in patients with hepatitis C virus-associated mixed cryoglobulinemia [9,10,14,15]. Clearly, the difference in BCR class expression could also contribute to differential signalling pathways since both the IgG and IgA BCR-classes were previously shown to boost proliferation and/or plasma cell commitment [32–36]. FCRL4 role in cell cycle inhibition is also supported by the concomitant overexpression of src-tyrosine kinases family members (HCK, FGR, LYN) and of the tyrosine phosphatase SHP1. Ehrhardt et al. have shown that FCRL4 activation results in tyrosine phosphorylation of ITIM domains by src-family kinases. This leads to SHP1 and SHP2 phosphatase recruitment and inhibition of BCR signaling [25,30]. Our transcriptome analysis showed that ten inhibitory receptors with ITIM domains are significantly overexpressed in FCRL4^+^ B cells compared with FCRL4^-^ cells, suggesting that they could also be involved in this process. However, among these receptors, *FCRL4* is the top upregulated gene in activated B cells at D4. The major role of FCRL4 in activated B cell inhibition is in agreement with the finding that HIV-associated B cell exhaustion can be overcome by using siRNAs against nine cell inhibitory receptors and that FCRL4 silencing has the strongest effect [14]. Moreover, analysis of FCRL family gene expression at the different steps of MBC differentiation to PCs [23] shows that FCRL4 is specifically expressed in activated B cells (S3 Fig). FCRL4 is a receptor for human IgA [16], it is possible that endogenous IgA secreted in the culture, mainly by FCRL4^-^ cells, activate this receptor inducing an inhibition of the proliferation of FCRL4^+^ cells (Fig 9). During immune response activation *in vivo*, this could also constitute a mechanism to control FCRL4^+^ cell expansion through IgA produced by PCs. We cannot exclude that exogenous antibodies (mouse IgG mAbs), used in our culture system for cell-stimulation or cell-sorting, could interfere and transduce inhibitory signals via the binding of their Fc fragment to the inhibitory receptor CD32b (FCGR). However, regarding an eventual role of the anti-histidine mAb used for cell-stimulation, we have shown that MBCs stimulation only with ODN resulted in the same decrease of FCRL4^+^ cells at D4 (Figs 4B and 1B). Moreover, when total cells were cultured from D4 to D7 in absence of exogenous mAbs, FCRL4^+^ cells shown the same delay in proliferation (Fig 7). We have verified that CD19 and CD27 mAbs used to sort MBCs were undetectable at D4 of culture.

Importantly, *in vitro*-generated FCRL4^+^ B cells can maintain FCRL4 expression or move back to the FCRL4^-^ compartment. At this stage, it remains unknown whether this could be interpreted as witnessing an intermediate and transient FCRL4^+^ differentiation stage, delaying proliferation and PC differentiation, or as a split into two different populations, notably entitled or not to PC differentiation. One subpopulation of purified FCRL4^+^ B cells was unable to differentiate into PCs and remained FCRL4 positive. The other FCRL4^+^ B cells differentiated into PCs with concomitant loss of FCRL4 expression. However, in accordance with their reduced cell proliferation, the number of PCs generated at D10 from FCRL4^+^ B cells was 4-fold lower compared with that from FCRL4^-^ B cells. Our results are in agreement with those by Tangye et al. showing that cell proliferation is mandatory for B cell differentiation into PCs [37]. Of note, in our culture system, FCRL4 expression was associated with a reduced number of cell divisions. As the two culture systems are quite similar, these data suggest that the poorly cycling B cells unable to differentiate into PCs described by Tangye et al. are *in vitro* generated FCRL4^+^ B cells.

Abnormal expression of FCRL4 and of other members of the FCRL family in B cells has been observed in many infectious and autoimmune diseases and could contribute to disorders leading to impaired immune or inflammatory response. Currently, the mechanisms involved in pathological FCRL4^+^ B cell generation are actively studied, but they remain elusive. Analysis of their normal counterpart could help understanding these mechanisms. As FCRL4^+^ cells are difficult to isolate *ex vivo*, our *in vitro* model could be of major interest for studying the biology of normal and pathological FCRL4+ cells.

## Supporting information

S1 Fig Expression of cytoplasmic IgH isotypes by D10 PCs generated by D4-sorted FCRL4^+^ and FCRL4^-^ cells. At D10 of culture, cells were labeled with mAbs against surface CD20 and CD138, then fixed, permeabilized and labeled with anti-human IgM, IgA and IgG mAbs. Results are the percentage of cytoplasmic (cy) IgM, IgA and IgG in CD20^-^ CD138^+^ PCs (mean ± SD of 4 experiments).
(PPTX)

S2 Fig Phenotype and cytoplasmic IgH isotypes expression by cells generated from D1-sorted FCRL4^+^ and FCRL4^-^ cells at each step of PC differentiation. **(A)** Percentage of FCRL4^+^ cells generated at D4, D7 and D10. **(B)** Percentage of prePBs generated at D4, of PBs at D7 and of PCs at D10. **(C)** Expression of cytoplasmic IgH isotypes by FCRL4^+^ and FCRL4^-^ generated from D1-sorted FCRL4^+^ cells and **(D)** generated from D1-sorted FCRL4^-^ cells at each step of PC differentiation. The analysis of FCRL4+ cells at D10 was not possible since this population accounts for less than 1% of total cells. **(E)** Expression of cytoplasmic IgH isotypes in D10 PCs. Results are the mean ± SD of three experiments. * The mean percentage is significantly different from D1-sorted FCRL4^+^ cells (*P* < 0.05).
(PPTX)

S3 Fig Gene expression analysis of FCRL family members during B cell differentiation, from naive B cells to PCs. Gene expression was evaluated using Affymetrix microarrays as previously described (19, 20, 28). Data are the mean signal from five purified primary naive B cell samples, four purified centroblast and centrocyte samples and five purified MBC samples. Then, five samples were obtained by *in vitro* differentiation of MBCs into CD20highCD38- activated B cells (five samples), prePBs (five samples), PBs (five samples), early PCs (five samples), and long-lived PCs (LLPCs, five samples). Data were analyzed with our bioinformatics platform GenomicScape (www.genomicscape.com) [23].
(PPTX)

S1 Table SAM supervised analysis to identify genes that are differentially expressed in D4 FCRL4^+^ and D4 FCRL4^-^ cells. (XLSX)

S2 Table Pathway enrichment analysis using Reactome. (XLSX)

S3 Table SAM supervised analysis to compare gene expression in D4 FCRL4^+^ cells and D0 FCRL4^-^ MBCs. (XLSX)

S4 Table Genes overexpressed in D4 FCRL4^+^ cells and in FCRL4^+^ MBCs puified from tonsils (Ehrhardt et al 2008). (XLSX)

S5 Table General characteristics of FCRL4^+^ cells generated in vitro, purified from tonsils, and from different tissues in various infectious and autoimmune diseases. (XLSX)

## References

[1] Sanz I, Wei C, Lee FE, Anolik J. Phenotypic and functional heterogeneity of human memory B cells. Semin Immunol. 2008;20: 67–82. doi: 10.1016/j.smim.2007.12.006 18258454 10.1016/j.smim.2007.12.006 PMC2440717

[2] Tangye SG, Tarlinton DM. Memory B cells: effectors of long-lived immune responses. Eur J Immunol. 2009;39: 2065–2075. doi: 10.1002/eji.200939531 19637202 10.1002/eji.200939531

[3] Tarlinton D, Good-Jacobson K. Diversity among memory B cells: origin, consequences, and utility. Science. 2013;341: 1205–1211. doi: 10.1126/science.1241146 24031013 10.1126/science.1241146

[4] Davis RS. Fc receptor-like molecules. Annu Rev Immunol. 2007;25: 525–560. doi: 10.1146/annurev.immunol.25.022106.141541 17201682 10.1146/annurev.immunol.25.022106.141541

[5] Hatzivassiliou G, Miller I, Takizawa J, Palanisamy N, Rao PH, Iida S, et al IRTA1 and IRTA2, novel immunoglobulin superfamily receptors expressed in B cells and involved in chromosome 1q21 abnormalities in B cell malignancy. Immunity. 2001;14: 277–289. 11290337 10.1016/s1074-7613(01)00109-1

[6] Falini B, Tiacci E, Pucciarini A, Bigerna B, Kurth J, Hatzivassiliou G, et al Expression of the IRTA1 receptor identifies intraepithelial and subepithelial marginal zone B cells of the mucosa-associated lymphoid tissue (MALT). Blood. 2003;102: 3684–3692. doi: 10.1182/blood-2003-03-0750 12881317 10.1182/blood-2003-03-0750

[7] Ehrhardt GR, Hsu JT, Gartland L, Leu CM, Zhang S, Davis RS, et al Expression of the immunoregulatory molecule FcRH4 defines a distinctive tissue-based population of memory B cells. J Exp Med. 2005;202: 783–791. doi: 10.1084/jem.20050879 16157685 10.1084/jem.20050879 PMC2212938

[8] Polson AG, Zheng B, Elkins K, Chang W, Du C, Dowd P, et al Expression pattern of the human FcRH/IRTA receptors in normal tissue and in B-chronic lymphocytic leukemia. Int Immunol. 2006;18: 1363–1373. doi: 10.1093/intimm/dxl069 16849395 10.1093/intimm/dxl069

[9] Moir S, Ho J, Malaspina A, Wang W, DiPoto AC, O'Shea MA, et al Evidence for HIV-associated B cell exhaustion in a dysfunctional memory B cell compartment in HIV-infected viremic individuals. J Exp Med. 2008;205: 1797–1805. doi: 10.1084/jem.20072683 18625747 10.1084/jem.20072683 PMC2525604

[10] Charles ED, Brunetti C, Marukian S, Ritola KD, Talal AH, Marks K, et al Clonal B cells in patients with hepatitis C virusâ€“associated mixed cryoglobulinemia contain an expanded anergic CD21low B-cell subset. Blood. 2011;117: 5425–5437. doi: 10.1182/blood-2010-10-312942 21421840 10.1182/blood-2010-10-312942 PMC3109715

[11] Bernard NJ. Rheumatoid arthritis: Are FcRL4+ B cells the next target for RA biologic therapy? Nat Rev Rheumatol. 2014;10: 127 doi: 10.1038/nrrheum.2014.8 24492385 10.1038/nrrheum.2014.8

[12] Yeo L, Lom H, Juarez M, Snow M, Buckley CD, Filer A, et al Expression of FcRL4 defines a pro-inflammatory, RANKL-producing B cell subset in rheumatoid arthritis. Ann Rheum Dis. 2015;74: 928–935. doi: 10.1136/annrheumdis-2013-204116 24431391 10.1136/annrheumdis-2013-204116 PMC4392201

[13] Zeng Z, Duan Z, Zhang T, Wang S, Li G, Mei Y, et al Association of FCRL4 polymorphisms on disease susceptibility and severity of ankylosing spondylitis in Chinese Han population. Clin Rheumatol. 2012;31: 1449–1454. doi: 10.1007/s10067-012-2028-y 22777505 10.1007/s10067-012-2028-y

[14] Kardava L, Moir S, Wang W, Ho J, Buckner CM, Posada JG, et al Attenuation of HIV-associated human B cell exhaustion by siRNA downregulation of inhibitory receptors. J Clin Invest. 2011;121: 2614–2624. doi: 10.1172/JCI45685 21633172 10.1172/JCI45685 PMC3127436

[15] Jelicic K, Cimbro R, Nawaz F, Huang DW, Zheng X, Yang J, et al The HIV-1 envelope protein gp120 impairs B cell proliferation by inducing TGF-β1 production and FcRL4 expression. Nat Immunol. 2013;14: 1256–1265. doi: 10.1038/ni.2746 24162774 10.1038/ni.2746 PMC3870659

[16] Wilson TJ, Fuchs A, Colonna M. Cutting edge: human FcRL4 and FcRL5 are receptors for IgA and IgG. J Immunol. 2012;188: 4741–4745. doi: 10.4049/jimmunol.1102651 22491254 10.4049/jimmunol.1102651 PMC3634363

[17] Ehrhardt GR, Hijikata A, Kitamura H, Ohara O, Wang JY, Cooper MD. Discriminating gene expression profiles of memory B cell subpopulations. J Exp Med. 2008;205: 1807–1817. doi: 10.1084/jem.20072682 18625746 10.1084/jem.20072682 PMC2525601

[18] Jourdan M, Caraux A, Caron G, Robert N, Fiol G, Reme T, et al Characterization of a transitional preplasmablast population in the process of human B cell to plasma cell differentiation. J Immunol. 2011;187: 3931–3941. doi: 10.4049/jimmunol.1101230 21918187 10.4049/jimmunol.1101230

[19] Jourdan M, Caraux A, De Vos J, Fiol G, Larroque M, Cognot C, et al An in vitro model of differentiation of memory B cells into plasmablasts and plasma cells including detailed phenotypic and molecular characterization. Blood. 2009;114: 5173–5181. doi: 10.1182/blood-2009-07-235960 19846886 10.1182/blood-2009-07-235960 PMC2834398

[20] Hartmann G, Krieg AM. Mechanism and function of a newly identified CpG DNA motif in human primary B cells. J Immunol. 2000;164: 944–953. 10623843 10.4049/jimmunol.164.2.944

[21] Maecker HT, Frey T, Nomura LE, Trotter J. Selecting fluorochrome conjugates for maximum sensitivity. Cytometry A. 2004;62: 169–173. doi: 10.1002/cyto.a.20092 15536642 10.1002/cyto.a.20092

[22] Jourdan M, Cren M, Schafer P, Robert N, Duperray C, Vincent L, et al Differential effects of lenalidomide during plasma cell differentiation. Oncotarget. 2016;7: 28096–28111. doi: 10.18632/oncotarget.8581 27057635 10.18632/oncotarget.8581 PMC5053712

[23] Kassambara A, Rème T, Jourdan M, Fest T, Hose D, Tarte K, et al GenomicScape: an easy-to-use web tool for gene expression data analysis. Application to investigate the molecular events in the differentiation of B cells into plasma cells. PLoS Comput Biol. 2015;11: e1004077 doi: 10.1371/journal.pcbi.1004077 25633866 10.1371/journal.pcbi.1004077 PMC4310610

[24] Eisen MB, Spellman PT, Brown PO, Botstein D. Cluster analysis and display of genome-wide expression patterns. Proc Natl Acad Sci USA. 1998;95: 14863–14868. 9843981 10.1073/pnas.95.25.14863 PMC24541

[25] Ehrhardt GR, Davis RS, Hsu JT, Leu CM, Ehrhardt A, Cooper MD. The inhibitory potential of Fc receptor homolog 4 on memory B cells. Proc Natl Acad Sci USA. 2003;100: 13489–13494. doi: 10.1073/pnas.1935944100 14597715 10.1073/pnas.1935944100 PMC263841

[26] Tusher VG, Tibshirani R, Chu G. Significance analysis of microarrays applied to the ionizing radiation response. Proc Natl Acad Sci USA. 2001;98: 5116–5121. doi: 10.1073/pnas.091062498 11309499 10.1073/pnas.091062498 PMC33173

[27] Jourdan M, Cren M, Robert N, Bollore K, Fest T, Duperray C, et al IL-6 supports the generation of human long-lived plasma cells in combination with either APRIL or stromal cell-soluble factors. Leukemia. 2014;28: 1647–1656. doi: 10.1038/leu.2014.61 24504026 10.1038/leu.2014.61

[28] Jöhrens K, Shimizu Y, Anagnostopoulos I, Schiffmann S, Tiacci E, Falini B, et al T-bet-positive and IRTA1-positive monocytoid B cells differ from marginal zone B cells and epithelial-associated B cells in their antigen profile and topographical distribution. Haematologica. 2005;90: 1070–1077. 16079106

[29] Amara K, Clay E, Yeo L, Ramsköld D, Spengler J, Sippl N, et al B cells expressing the IgA receptor FcRL4 participate in the autoimmune response in patients with rheumatoid arthritis. J Autoimmun. 2017 doi: 10.1016/j.jaut.2017.03.004 28343748 10.1016/j.jaut.2017.03.004 PMC5473332

[30] Daëron M, Jaeger S, Pasquier Du L, Vivier E. Immunoreceptor tyrosine-based inhibition motifs: a quest in the past and future. Immunol Rev. 2008;224: 11–43. doi: 10.1111/j.1600-065X.2008.00666.x 18759918 10.1111/j.1600-065X.2008.00666.x

[31] Sohn HW, Krueger PD, Davis RS, Pierce SK. FcRL4 acts as an adaptive to innate molecular switch dampening BCR signaling and enhancing TLR signaling. Blood. 2011;118: 6332–6341. doi: 10.1182/blood-2011-05-353102 21908428 10.1182/blood-2011-05-353102 PMC3236118

[32] Waisman A, Kraus M, Seagal J, Ghosh S, Melamed D, Song J, et al IgG1 B cell receptor signaling is inhibited by CD22 and promotes the development of B cells whose survival is less dependent on Ig alpha/beta. J Exp Med. 2007;204: 747–758. doi: 10.1084/jem.20062024 17420268 10.1084/jem.20062024 PMC2118546

[33] Pogue SL, Goodnow CC. Gene dose-dependent maturation and receptor editing of B cells expressing immunoglobulin (Ig)G1 or IgM/IgG1 tail antigen receptors. J Exp Med. 2000;191: 1031–1044. 10727464 10.1084/jem.191.6.1031 PMC2193121

[34] Martin SW, Goodnow CC. Burst-enhancing role of the IgG membrane tail as a molecular determinant of memory. Nat Immunol. 2002;3: 182–188. doi: 10.1038/ni752 11812996 10.1038/ni752

[35] Duchez S, Amin R, Cogné N, Delpy L, Sirac C, Pascal V, et al Premature replacement of mu with alpha immunoglobulin chains impairs lymphopoiesis and mucosal homing but promotes plasma cell maturation. Proc Natl Acad Sci USA. 2010;107: 3064–3069. doi: 10.1073/pnas.0912393107 20133609 10.1073/pnas.0912393107 PMC2840347

[36] Laffleur B, Denis-Lagache N, Péron S, Sirac C, Moreau J, Cogné M. AID-induced remodeling of immunoglobulin genes and B cell fate. Oncotarget. 2014;5: 1118–1131. doi: 10.18632/oncotarget.1546 24851241 10.18632/oncotarget.1546 PMC4012742

[37] Tangye SG, Avery DT, Hodgkin PD. A division-linked mechanism for the rapid generation of Ig-secreting cells from human memory B cells. J Immunol. 2003;170: 261–269. 12496408 10.4049/jimmunol.170.1.261

