# Supplementary material for: Characterization of human FCRL4-positive B cells
Source: PLoS One. 2017 Jun 21;12(6):e0179793. doi: 10.1371/journal.pone.0179793 (PMC5479562; doi:10.1371/journal.pone.0179793)
Supplement: S1 Fig — At D10 of culture, cells were labeled with mAbs against surface CD20 and CD138, then fixed, permeabilized and labeled with anti-human IgM, IgA and IgG mAbs. Results are the percentage of cytoplasmic (cy) IgM, IgA and IgG in CD20- CD138+ PCs (mean ± SD of 4 experiments). (PPTX) [file pone.0179793.s001.pptx]

## Slide 1
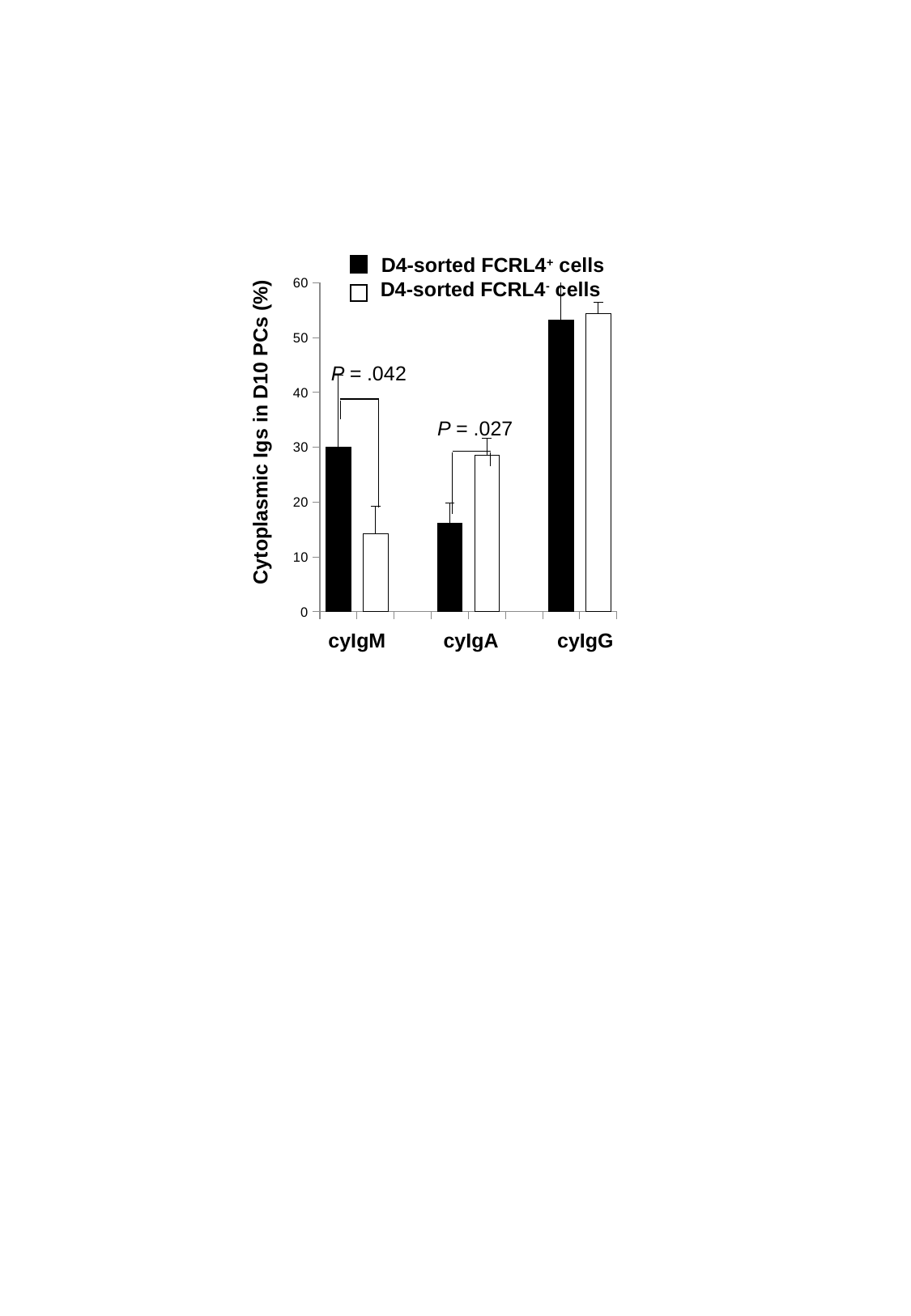

D4-sorted FCRL4+ cells
D4-sorted FCRL4- cells
### Chart
| Category | |
|---|---|P = .042
P = .027
Cytoplasmic Igs in D10 PCs (%)
cyIgM
cyIgA
cyIgG
