# Supplementary material for: Characterization of human FCRL4-positive B cells
Source: PLoS One. 2017 Jun 21;12(6):e0179793. doi: 10.1371/journal.pone.0179793 (PMC5479562; doi:10.1371/journal.pone.0179793)
Supplement: S2 Fig — (A) Percentage of FCRL4+ cells generated at D4, D7 and D10. (B) Percentage of prePBs generated at D4, of PBs at D7 and of PCs at D10. (C) Expression of cytoplasmic IgH isotypes by FCRL4+ and FCRL4- generated from D1-sorted FCRL4+ cells and (D) generated from D1-sorted FCRL4- cells at each step of PC differentiation. The analysis of FCRL4+ cells at D10 was not possible since this population accounts for less than 1% of total cells. (E) Expression of cytoplasmic IgH isotypes in D10 PCs. Results are the mean ± SD of three experiments. * The mean percentage is significantly different from D1-sorted FCRL4+ cells (P < 0.05). (PPTX) [file pone.0179793.s002.pptx]

## Slide 1
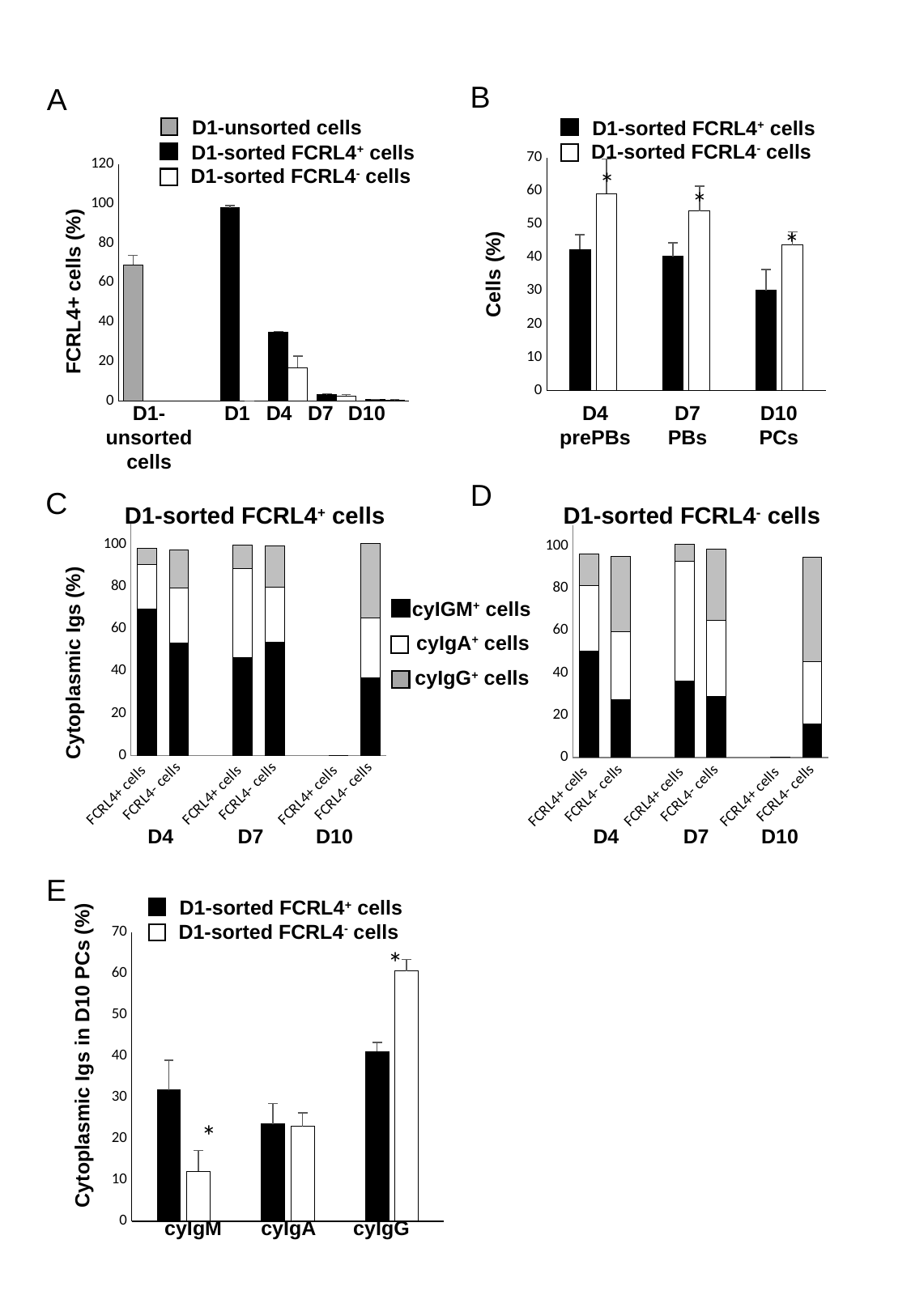

B
A
D1-unsorted cells
D1-sorted FCRL4+ cells
D1-sorted FCRL4- cells
D1-sorted FCRL4+ cells
D1-sorted FCRL4- cells
### Chart
| Category | | |
|---|---|---|
| D4 prePBs | 42.43 | 59.215 |
| D7 PBs | 40.35 | 53.965 |
| D10 PCs | 30.035 | 43.78 |
### Chart
| Category | | |
|---|---|---|
| D1-unsorted cells | 68.905 | None |
| | None | None |
| D1 | 98.0 | 0.0 |
| D4 | 34.89 | 16.955 |
| D7 | 3.1 | 2.42 |
| D10 | 0.56 | 0.385 |*
*
*
Cells (%)
FCRL4+ cells (%)
D4
prePBs
D7
PBs
D10
PCs
D1
D4
D7
D10
D1-
unsorted cells
D
C
D1-sorted FCRL4+ cells
D1-sorted FCRL4- cells
### Chart
| Category | | | |
|---|---|---|---|
| FCRL4+ cells | 69.47 | 21.16 | 7.564999999999997 |
| FCRL4- cells | 53.35 | 26.17 | 18.035 |
| | None | None | None |
| FCRL4+ cells | 46.505 | 42.065 | 10.975 |
| FCRL4- cells | 53.73 | 25.95 | 19.655 |
| | None | None | None |
| FCRL4+ cells | 0.0 | 0.0 | 0.0 |
| FCRL4- cells | 36.895 | 28.415 | 35.12 |
### Chart
| Category | | | |
|---|---|---|---|
| FCRL4+ cells | 50.395 | 31.185 | 14.745 |
| FCRL4- cells | 27.275 | 32.47 | 35.515 |
| | None | None | None |
| FCRL4+ cells | 36.23 | 56.485 | 8.215 |
| FCRL4- cells | 29.075 | 35.985 | 33.415 |
| | None | None | None |
| FCRL4+ cells | 0.0 | 0.0 | 0.0 |
| FCRL4- cells | 16.035 | 29.45 | 49.38 |cyIGM+ cells
cyIgA+ cells
Cytoplasmic Igs (%)
cyIgG+ cells
D4
D7
D10
D4
D7
D10
E
D1-sorted FCRL4+ cells
D1-sorted FCRL4- cells
### Chart
| Category | | |
|---|---|---|
| IgM | 31.805 | 12.06 |
| IgA | 23.52 | 22.96 |
| IgG | 41.125 | 60.595 |*
Cytoplasmic Igs in D10 PCs (%)
*
cyIgM
cyIgA
cyIgG
