# Supplementary figures and images for: Characterization of human FCRL4-positive B cells
Source: PLoS One. 2017 Jun 21;12(6):e0179793. doi: 10.1371/journal.pone.0179793 (PMC5479562; doi:10.1371/journal.pone.0179793)

## Slide 1
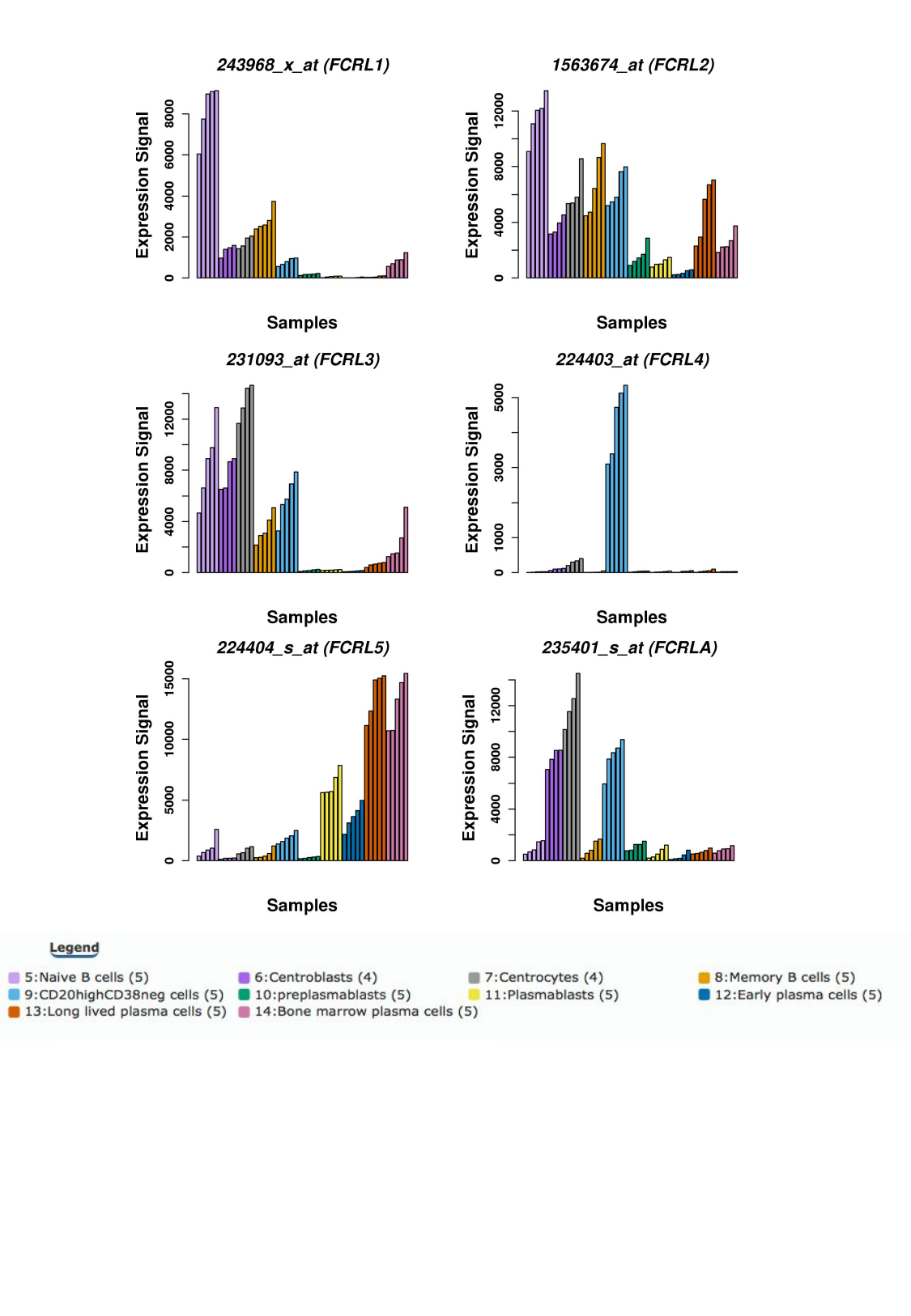

Supplement: S3 Fig — Gene expression was evaluated using Affymetrix microarrays as previously described (19, 20, 28). Data are the mean signal from five purified primary naive B cell samples, four purified centroblast and centrocyte samples and five purified MBC samples. Then, five samples were obtained by in vitro differentiation of MBCs into CD20highCD38- activated B cells (five samples), prePBs (five samples), PBs (five samples), early PCs (five samples), and long-lived PCs (LLPCs, five samples). Data were analyzed with our bioinformatics platform GenomicScape (www.genomicscape.com) [23]. (PPTX) [file pone.0179793.s003.pptx]
